# Metastability of Neuronal Dynamics during General Anesthesia: Time for a Change in Our Assumptions?

**DOI:** 10.3389/fncir.2017.00058

**Published:** 2017-08-25

**Authors:** Andrew E. Hudson

**Affiliations:** Department of Anesthesiology and Critical Care Medicine, David Geffen School of Medicine, University of California, Los Angeles Los Angeles, CA, United States

**Keywords:** metastability, isoflurane, rats, Sprague-Dawley, attractor networks, attractor dynamics

## Abstract

There is strong evidence that anesthetics have stereotypical effects on brain state, so that a given anesthetic appears to have a signature in the electroencephalogram (EEG), which may vary with dose. This can be usefully interpreted as the anesthetic determining an attractor in the phase space of the brain. How brain activity shifts between these attractors in time remains understudied, as most studies implicitly assume a one-to-one relationship between drug dose and attractor features by assuming stationarity over the analysis interval and analyzing data segments of several minutes in length. Yet data in rats anesthetized with isoflurane suggests that, at anesthetic levels consistent with surgical anesthesia, brain activity alternates between multiple attractors, often spending on the order of 10 min in one activity pattern before shifting to another. Moreover, the probability of these jumps between attractors changes with anesthetic concentration. This suggests the hypothesis that brain state is metastable during anesthesia: though it appears at equilibrium on short timescales (on the order of seconds to a few minutes), longer intervals show shifting behavior. Compelling evidence for metastability in rats anesthetized with isoflurane is reviewed, but so far only suggestive hints of metastability in brain states exist with other anesthetics or in other species. Explicit testing of metastability during anesthesia will require experiments with longer acquisition intervals and carefully designed analytic approaches; some of the implications of these constraints are reviewed for typical spectral analysis approaches. If metastability exists during anesthesia, it implies degeneracy in the relationship between brain state and effect site concentration, as there is not a one-to-one mapping between the two. This degeneracy could explain some of the reported difficulty in using brain activity monitors to titrate drug dose to prevent awareness during anesthesia and should force a rethinking of the notion of depth of anesthesia as a single dimension. Finally, explicit incorporation of knowledge of the dynamics of the brain during anesthesia could offer better depth of anesthesia monitoring.

## Introduction

General anesthesia is a pharmacologically-induced, reversible state of unarousable unresponsiveness. As in other states of unconsciousness, such as absence seizure or the vegetative state, the brain is not necessarily electrically quiescent during general anesthesia. From the earliest days of electroencephalography (EEG), Gibbs et al. (1937) reported stereotypic shifts with increasing doses of ether anesthesia: as doses increase, rhythms slow and increasing power is found at low frequencies of the EEG. Much of the subsequent work on systems and circuit level mechanisms of anesthesia has focused on defining the corresponding power spectrum signatures for different anesthetic agents (Purdon et al., 2015; Akeju et al., 2016a,b; Pavone et al., 2016).

As the field has adopted more sophisticated analytic approaches to electrical activity during anesthesia, including the prominent use of the spectrogram to track the time evolution of the frequency content of brain signals, groups have begun to take interest not only in the steady state response to anesthetic drugs, but also to the ways in which the brain transitions into (Ishizawa et al., 2016) or out of those steady state responses (Chander et al., 2014; Hudson et al., 2014). One persistent feature in all of these studies is that brain states spontaneously shift. Spontaneous changes in network state can even occur during quiet wakefulness with no anesthetic in mice (Zagha et al., 2013; McCormick et al., 2015), which has usually been attributed to shifts in subcortical arousal-related nuclei (Moruzzi and Magoun, 1949; Steriade et al., 1993). It should perhaps come as no surprise that state switching occurs during anesthesia in the absence of a change of anesthetic or stimulus (Hudson et al., 2014), and the shifts between states may even prove predictive of features of a patient’s postoperative recovery (Chander et al., 2014).

As dynamical models of brain state increase in sophistication and utility (Breakspear, 2017), the time seems right to make the dynamics of brain activity during anesthesia a formal topic of study in and of itself. That is, how are brain activity patterns connected to one another? Are shifts between them gradual or sudden? Predictable or random? Are they dictated solely by the concentration of anesthesia in the brain or is there a component of state dependence? These questions will necessitate some common terminology and may even drive some changes in experimental design and analysis for studies of anesthetics as a systems level. This has implications for clinical practice: while I may be able to predict from ongoing EEG that a patient will not be aware at this moment in time, I would also like to know the likelihood that my patient’s brain will shift to a state that is aware before it happens, so I can prevent it.

## Attractors, Phase Spaces and Stability in Dynamical Systems

To begin, it is worthwhile to establish a vocabulary for talking about the behavior of the brain over time. Imagine a phase space: a description of the brain that captures all of the dynamical behaviors of interest, e.g., a multidimensional space where the activity of each neuron, perhaps as a firing rate, mean field voltage, or power spectral density, could be plotted on a separate axis. The study of the how the brain’s activity changes in time would then amount to mapping the trajectories that the brain takes through phase space.

**Figure 1:**
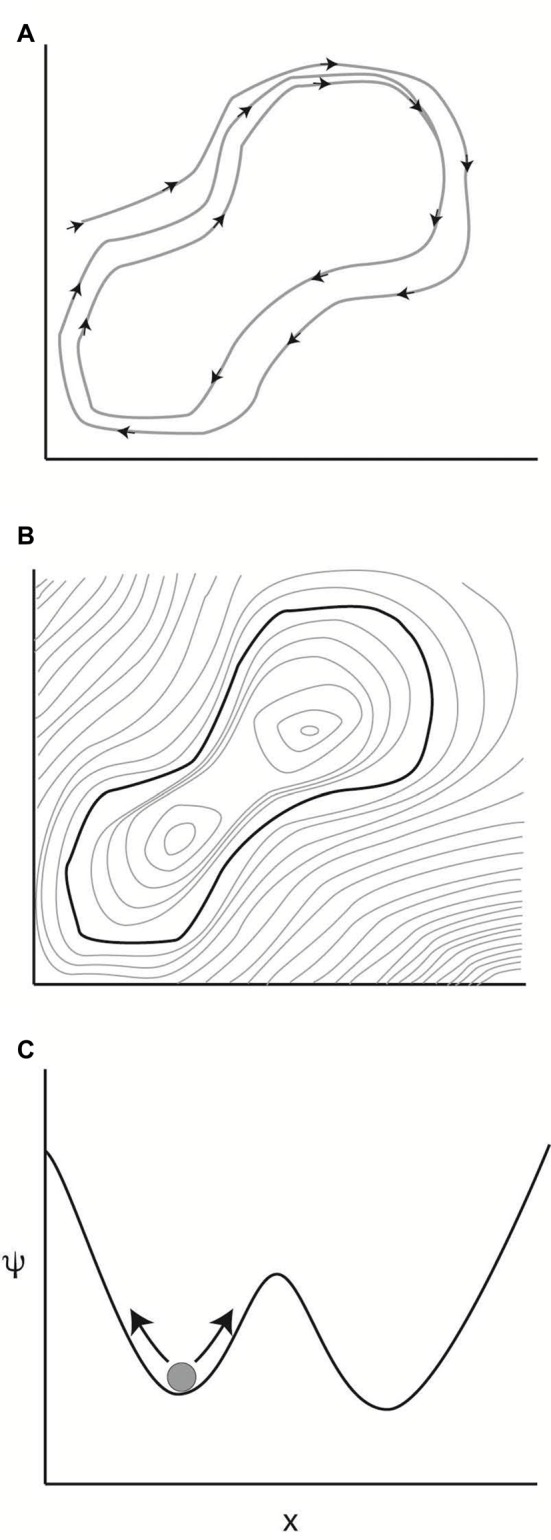
Attractors in dynamical systems. **(A)** A dynamical system evolving in time in a two dimensional phase space. Over time, the trajectory of the system closes on itself into an orbit. If the system is restarted in the neighborhood of this orbit, it tends to move closer to the described orbit. This convergence in the long time limit makes this orbit an attractor of the system. **(B)** The attractor can be thought of as a minimum on a potential surface. Light gray lines indicate uniform values of the potential function, like altitude in a topographic map. **(C)** One of the simplest systems capable of multistability.

The existence of certain stable signatures associated with anesthetic administration, such as the delta rhythm with volatile anesthetics (Purdon et al., 2015) or the frontalization of posterior alpha rhythms (Tinker et al., 1977), suggests that certain activity patterns can reverberate through brain circuits in a persistent pattern, which in phase space would be seen as a trajectory that closes on itself in an orbit. If the brain tends towards developing that trajectory, such that if the brain starts off on a trajectory that is close to that orbit, it remains close to the orbit later in time, the trajectory is described as an attractor in the system (Figure 1A).

To understand stability in this context, it is helpful to imagine the phase space as a two dimensional projection of a potential landscape: if the potential involved in each state were plotted on a vertical axis, the attractor would correspond to the bottom of a valley in the potential landscape (Figure 1B). Points at higher potential would tend downhill, and the system will eventually settle into the attractor’s dynamics. If the attractor is at the global minimum on the potential landscape, the system is stable and will not deviate from the trajectory defined by that attractor (A dynamical system may have more than one attractor. As an example, coupled oscillators tend to synchronize themselves either at the same phase or 180° out of phase. The slope of the potential landscape will determine which attractor wins out depending upon the starting state of the system).

## Noise and Metastability in Dynamical Systems

The observation of stability may depend on the timescale of observation. A system may appear to be at a stable equilibrium state—that is, in the basin of a stable attractor—when examined on short timescales but on longer time scales it might jump between several different attractors. This behavior, with multiple, well-separated timescales, is described as metastability (Bovier, 2006).

In modeling metastability, it is useful to consider a system with more than one stable attractor and additive noise. In the simplest case, this would be a particle diffusing back and forth in one dimension, with dynamics defined by a two potential well (Figure 1C). Here, the phase space is one dimensional, and the value of the parameter *x* is indicated on the abscissa, with the potential function ψ plotted on the ordinate in Figure 1C. Two attractors for the system are indicated by the two minima in the potential function. The current state of the system is indicated by the ball in the left local minimum of the potential function. The arrows on either side of the ball indicate that the system is being continually perturbed by a noise input. As the system evolves in time, the probability is high that it will remain in the starting well, so that, on short timescales, the particle will converge towards the attractor defined by the potential landscape. However, for certain values of noise input, the system will occasionally jump over the boundary to the other well.

The system will thus have two vastly different time scales and exhibit metastability. This simple model suggests that one way of thinking of metastability is a finite state Markov chain, where each state corresponds to an attractor in the system, with exponentially small transition probabilities (Bovier, 2006). The Markovian assumption is simply that the transition probabilities between states depends only upon the current state, with no history dependence.

Metastable systems will spend most of their time near attractors, with rare, rapid transitions between them. This implies that, for long observation periods the system will be clustered around the attractors, and that dwell times near an attractor will be long relative to transition times between attractors.

It is worth noting that some authors define multistability as the case of a noise perturbed system with multiple stable attractors and metastability as the case of a system that is not quite stable because the time evolution of the system never goes to zero (Kelso, 2012), that is, a metastable system is intrinsically unstable, whereas a multistable system would be stable if you could decrease noise below the threshold that allows transitions between attractors (This is more or less a question of whether the noise element is intrinsic to the system or can be separated out, and I will neglect it here).

## Attractors in Brain Dynamics During Anesthesia

**Figure 2:**
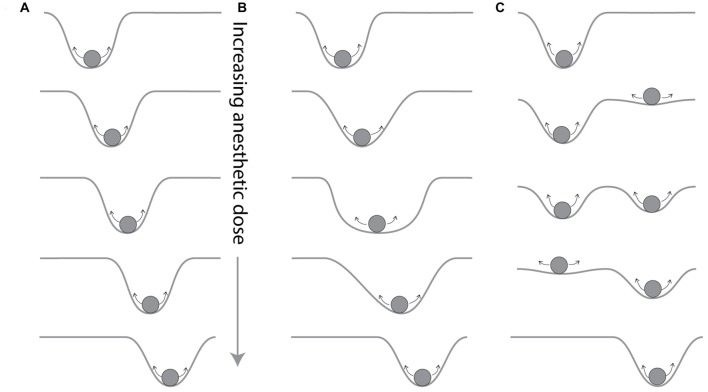
Possible dependencies of attractors on anesthetic concentration. The brain appears to engage in stereotyped behavior when a given anesthetic concentration is delivered to the brain, suggesting that a given drug and dose establish an attractor for the brain, here imagined as a particle on a potential surface that is constantly disturbed by noise, indicated by small arrows, as in Figure 1C. The way in which these attractors change as the anesthetic concentration changes has not been well characterized. **(A)** The attractor could move continuously as the anesthetic is increased. **(B)** The width of the well, which defines it’s stability, as well as its location, could shift. Or **(C)** the nature of the attractor landscape could qualitatively change, with the appearance of a new attractor that could coexist with the original attractor for some anesthetic concentrations.

The presence of ongoing, stereotypic patterns in EEG with different anesthetics, reported as brain “signatures” of anesthetics (Purdon et al., 2015), is consistent with the presence of stable attractors in brain dynamics. The search for a signature of each anesthetic assumes that there is a distinct underlying attractor is determined by the anesthetic and its effect-site concentration. As the dose of anesthetic increases, attractors could shift their location in phase space in a continuous fashion (Figure 2A), could vary their width (Figure 2B), or could change in number or some other qualitative fashion (Figure 2C). Qualitative changes, such as changes in the number of attractors with changes in anesthetic dose, correspond to qualitative shifts in behavior analogous to phase transitions in thermodynamics models, and can be produced from perturbing population behavior models of cortex (Steyn-Ross et al., 1999, 2004). Thus, it is reasonable to propose the design of depth of anesthesia monitors that detect the presence or features of a particular attractor (Walling and Hicks, 2006).

## Electrophysiology Methods

This article includes reanalysis of a previously published dataset (Hudson et al., 2014), which described in detail the methods for the simultaneous acquisition of multiple channels of cortical and thalamic local field potentials (LFPs) from five Sprague-Dawley rats spontaneously breathing isoflurane in 100% oxygen. All experiments were carried out in accordance with the National Institutes of Health *Guide for the Care and Use of Laboratory Animals* and approved by the Rockefeller University’s Institutional Animal Care and Use Committee.

In brief, all experiments were conducted acutely. Animals were induced with 3% isoflurane, a craniotomy opened, and a custom linear microarray (Alpha Omega, Alpharetta, GA, USA) was placed stereotacticaly to acquire eight channels of LFP from anterior cingulate and retrosplenial cortex and the hippocampus and centrolateral nucleus of the thalamus (total of 24 channels). Signals were sampled at 40 kHz and streamed to disk by a multichannel op-amp system (Plexon). LFPs were extracted with an acausal fourth-order Butterworth filter, using a low-pass frequency of 500 Hz to minimize phase distortion and then downsampled to 1 kHz. Delivered anesthetic concentration was maintained for at least 60 min of acquisition, beginning at 1.75% isoflurane (sufficient to produce burst suppression). The delivered isoflurane concentration was then reduced stepwise by 0.25% and another 60 min of data acquired. At 0.75% delivered isoflurane, all animals began moving spontaneously; at that point the delivered anesthetic was then increased and other experiments completed.

## Metastability Occurs During Anesthesia

**Figure 3:**
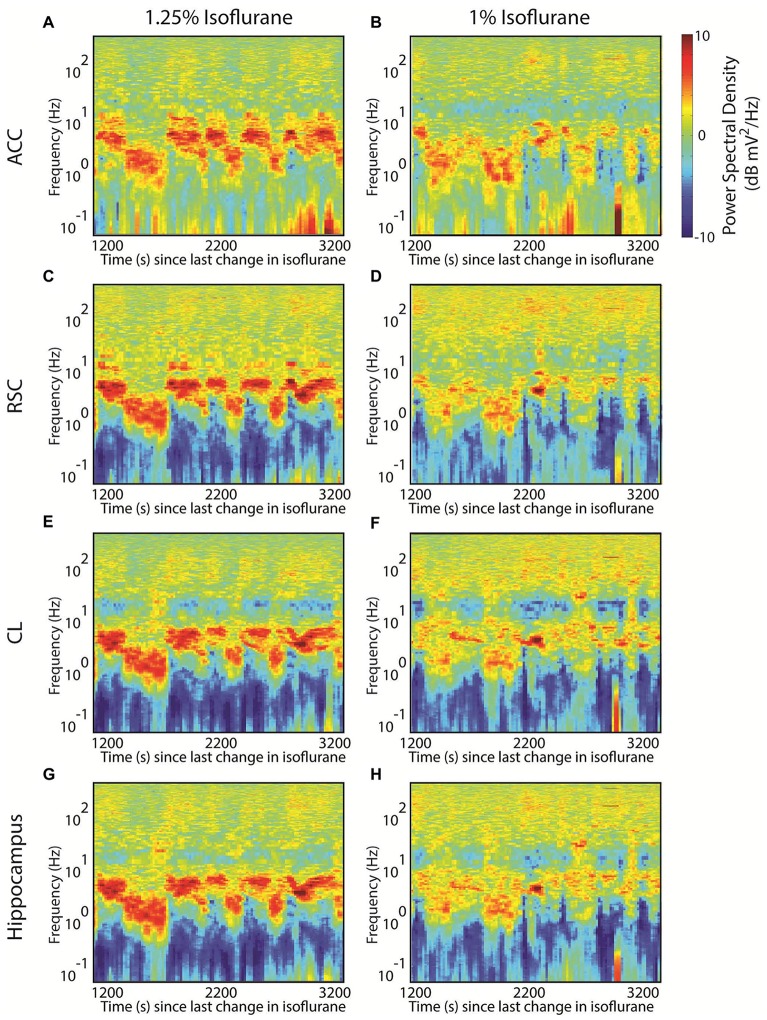
Evidence for multistability during isoflurane anesthesia in the rat. **(A)** The spectrogram from an local field potential (LFP) recorded in deep anterior cingulate cortex of a rat breathing 1.25% isoflurane. Deviations from the overall mean power spectrum for that animal are indicated as decibel differences according to the color scale. Plotted data begin 20 min after isoflurane was decreased from 1.5% to 1.25%, and the duration of the plot is 2000 s. Note the frequent alternation between power spectra dominated by peaks at ~6 Hz and ~2 Hz. **(B)** Data from anterior cingulate, beginning 20 min after the isoflurane was decreased from 1.25% to 1% isoflurane. Here short lived examples of both the ~6 Hz and ~2 Hz peaks exist, but there are instances of more high frequency power (above 100 Hz), especially later in the recording. **(C,D)** The corresponding spectrograms from simultaneously recorded retrosplenial cortex; **(E,F)** from central lateral nucleus of the thalamus; **(G,H)** and from hippocampus.

Interestingly, we have previously demonstrated that, at least in rats anesthetized with isoflurane, the LFP demonstrates metastability (Hudson et al., 2014). Namely, during equilibrium administration of isoflurane, the LFP exhibited a stable spectral signature, often for 8–12 min, before spontaneously shifting to a different signature. This can be seen in the shifts between two prominent spectral peaks; one centered at roughly 6 Hz and the other at roughly 2 Hz in the spectrogram in both anterior cingulate (Figure 3A), retrosplenial cortex (Figure 3C), thalamus (Figure 3E) and hippocampus (Figure 3G). These shifts occurred in the absence of any sensory stimulation or change in delivered anesthetic concentration. Several such signatures were possible at all tested levels of isoflurane, spanning from burst suppression to the return of spontaneous movement. Moreover, several signatures could exist at more than one anesthetic concentration: note that peaks at similar frequencies also occur at 1% isoflurane but the dwell time that the system spends at each frequency is shorter (Figures 3B,D,F,H) (Also note the appearance of more high frequency power at certain time points at 1% isoflurane, suggesting that another attractor may be appearing at this concentration). The presence of peaks at both frequencies at both anesthetic concentrations implies that the relationship between anesthetic effect site concentration and brain state is degenerate. Note also that the change in dwell times—or, alternatively, transition probabilities—with anesthetic concentration suggests that the stability of a particular feature can change as a function of anesthetic.

**Figure 4:**
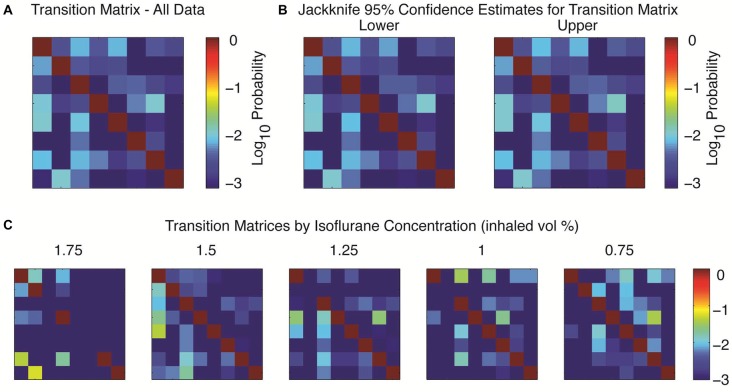
Transition probabilities between attractors. **(A)** The spectral data were clustered across all five animals (see text) and modeled as a Markov chain to calculate the transition matrix between each cluster. Here the probability of the system starting in the given row and on the next independent time step transitioning to the cluster in the indicated column are indicated by the log color axis, with a minimum probability assigned as 10^−3^. **(B)** Upper and lower bounds on the transition matrices, 95% confidence interval by jackknife over the five animals. **(C)** The transition probabilities depend upon the delivered anesthetic concentration. Not all clusters occurred at all anesthetic concentrations; for non-observed clusters all values in its row and column are assigned a probability of 10^−3^.

To study transition properties between states we first sought to combine data from five different rats, as a single hour of data acquisition might only yield 5–10 examples of transitions between observed states (e.g., Figure 3A). Following the approach first detailed in Hudson et al. (2014), the spectral signatures could be abstracted across animals by using principal components analysis and then clustered into eight clusters with a k-means algorithm using a Euclidean distance metric in the space defined by the first three principal components. This small number of clusters strongly suggests the existence of consistent attractors in brain dynamics (see Hudson et al., 2014 for further details on clustering; similar clusters were derived with multiple different clustering algorithms). The resulting shifts between attractors were modeled as the transition probabilities of a Markov chain, which revealed that the transition probabilities between states were low and non-uniform (Figure 4A). Consistent with metastable behavior, at any given time the probability of remaining in a given cluster was much higher than the probability of transitioning to any other cluster; note that the probabilities are plotted on a log scale, so the probabilities of transitioning between clusters are orders of magnitude smaller than the probability of remaining in the same cluster. In other words, brain dynamics during isoflurane anesthesia are “sticky”. This transition matrix was computed by collapsing over five separate concentrations of isoflurane (1.75% dropping to 0.75% in 0.25% increments). The resulting estimates were relatively stable across animals, as shown in the jackknife estimate of 95% confidence limits shown in Figure 4B.

The probability of each cluster occurring, and the particular transition probabilities depended upon the delivered concentration of anesthetic. Not all clusters were present at all concentrations, but some clusters occurred at every anesthetic concentration (Figure 4C). This reinforces the earlier, single trial impression of degeneracy between the brain state and drug dose. Similar transition matrices could be derived for each animal (data not shown), so the individual clusters are not specific for particular animals, and the results are reflective of the dynamics for each individual.

## Hypothesis: Is Metastability During Anesthesia A Generic Phenomenon?

It is worth explicitly investigating the prevalence of metastability during general anesthesia with different agents and in different species. It seems unlikely based upon the varying time profiles published in single-subject time-frequency spectrograms in the literature that the existence of multiple attractors with low transition probabilities between them is an isolated occurrence with isoflurane in rats. Certainly, the examination of time-frequency plots published from data acquired in rats anesthetized with sevoflurane (Guidera et al., 2017), monkeys given propofol (Ishizawa et al., 2016) and humans given a range of anesthetics (Chander et al., 2014) suggests that rich dynamics exist with multiple anesthetic regimens and in multiple species. Yet, none of these reports explicitly studied the ongoing transitions in brain state over long time intervals, so the chance to comment on the ubiquity of metastability or lack thereof currently remains untested. There are two possible alternatives: (1) brain activity demonstrates no attractors during anesthesia but will explore a phase space randomly; and (2) there is only a single stable attractor during the anesthetized state, which might depend upon the anesthetic concentration, so that the only requirement for knowing the brain state in a long time limit is the equilibrium concentration of delivered anesthetic.

The persistence of specific anesthetic signatures across subjects argues that attractors in the anesthetized state exist. Moreover, it seems exceptionally unlikely that random exploration of a high-dimensional state space would be compatible with the generally rapid recovery of consciousness after anesthetic exposure. If we make the reasonable assumption that only a finite region of a phase space is compatible with consciousness (Babloyantz et al., 1985), it is possible to model the recovery of consciousness after anesthesia process as a random walk through phase space. With increasing dimensions in the phase space, it is easily shown that recovery times will rapidly exceed those observed in practice (Hudson et al., 2014). The observed, finite recovery times in the majority of anesthetics argues that the intrinsic dimensionality of the space that the brain explores must be significantly smaller than a random walk through a high dimensional state space. Given that brain dynamics appear to be governed by multiple attractors during anesthesia, discontinuous jumps between attractors should probably be the expectation, rather than the exception, to transitions during recovery of consciousness.

While it is possible that some anesthetic concentrations may produce only a single attractor, there is no reason* a priori* to assume that this will be true generically across behavioral states. Sleep architecture, for example, consists of multiple attractors that are persistent for tens of minutes (Kishi et al., 2008), similar to the duration of attractors present observed in the rat. Finally, the presence of multiple patterns of recovery in the EEG after anesthesia (Lee et al., 2011; Hight et al., 2014) strongly suggests that the brains traverse more than one route through state space to recover consciousness, suggesting that different attractors may dominate an individual’s anesthetic period depending upon factors that remain to be elucidated.

While Hudson et al. (2014) assumed a Markov process for the shifts between attractors, this was intended to be as agnostic as possible: there has been essentially no work done on the distribution of dwell times at a particular attractor. While a Markov chain would produce an exponential dwell time distribution, some sleep stages have durations that approximate an exponential distribution while others approximate a power law (Kishi et al., 2008), suggesting that state dependence in state transitions during anesthesia could occur and should be empirically tested. Alternatively, the transitions could be a predictable, rather than stochastic phenomenon, given more observations of the state transitions. This will likely require much longer data sets at fixed anesthetic concentrations than are currently available.

## Discussion

### Detecting Metastability: Implications for Experimental Design

Given that the signatures associated with a given anesthetic can vary with drug concentration, a pharmacologic steady state must be achieved to attribute metastability to a system. Thus, the decision to acquire data for relatively long intervals (60 min at each concentration) was crucial for the identification of the identification of metastability in Hudson et al. (2014). More studies with long acquisition times and minimal external stimulation will be needed to address this issue. It would seem that the Reconstructing Human Consciousness and Cognition Study described in this issue (Maier et al., 2017) would provide an ideal opportunity to address this question in humans.

### Detecting Metastability: Implications for Data Analysis

The possible presence of long timescale dynamics in experiments using anesthesia should be expressly considered when designing a data analysis approach. Special care should be given to averaging across time and across subjects. Attempts to detect metastability in brain behavior during anesthesia should first look for state transitions within single subjects, and then pool state transition data across subjects. Given the propensity for researchers to use spectral methods on brain data, I will address the potential pitfalls with spectral analysis techniques specifically.

**Figure 5:**
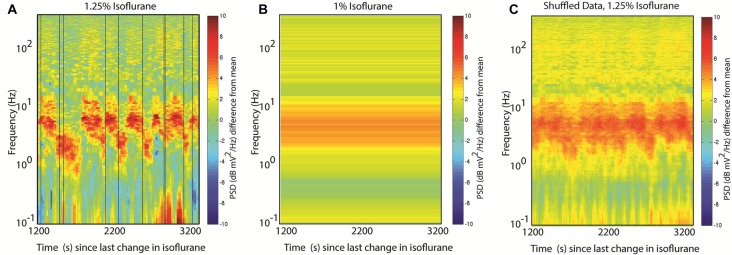
Spectral analysis challenges with metastability. **(A)** Randomly chosen analysis times. The same time-frequency data shown in Figure 3A. Vertical black lines indicate 10 randomly chosen times during the acquired interval.** (B)** Welch’s method fails with nonstationary inputs: the power spectrum estimate obtained by averaging the 30 s of data on either side of each of the random times shown in **(A)**. Compared with **(A)**, the two peaks at 6 Hz and 2 Hz are blended into a single wide peak. **(C)** Averaging spectrograms across animals with similar brain dynamics will again abolish dynamical features. Here the same random time points from **(A)** are used to create surrogate spectrograms by using the time to start the data acquisition and wrap around to the start of the spectrogram once the end is reached; the 10 resulting spectrograms are then averaged together, abolishing most of the alternating features.

Many spectral analysis approaches utilize a version of Welch’s method, which takes multiple, independent spectral estimates from different time windows and averages them together to obtain a smoother, more reliable estimate than is possible from a single window. Obviously, if the different windows capture the dynamics from more than one attractor, the different behaviors will be blurred together. In this instance, the reported state might reflect the average activity during the anesthetic administration, and yet in alternating between two states the brain may never actually exhibit the reported mean spectrum (Figures 5A,B). Another pitfall to keep in mind: data-driven artifact rejection approaches might discard a less-frequently observed pattern, biasing the reported spectral activity to the most frequently observed state.

A subtler, but just as confounding issue, can arise from averaging spectrograms across subjects. There is no reason to assume that the transitions between attractors occur at stereotyped times; rather, with metastable systems the state transitions appear to be stochastic events. As a result, averaging across subjects can similarly obscure state transitions by blurring two patterns together as they are distributed in time. In Figure 5C this is illustrated by constructing 10 surrogate datasets that have the same mean spectrogram and transition matrices by shuffling the start time of the dataset, and then averaging the resulting pseudo-data together. Notice the behavior of the alternating peaks is obscured in the resulting plot.

### Predictions of Metastability: Dynamical Timescales vs. Pharmacologic Equilibration

The presence of low transition probabilities between the observed brain states suggests that brain dynamics can themselves have timescales that are potentially separable from pharmacologic timescales, which may have implications for the timescale of recovery of consciousness after anesthesia. Although recovery was traditionally thought of as a passive process due to pharmacokinetics, the existence of neural inertia (Friedman et al., 2010), or the difference in EC50 between induction and emergence from anesthesia, could be consistent with a longer dynamical timescale to the process of brain state shifting during recovery. That is, a dynamical timescale longer than the pharmacokinetic timescale for a drug would, in itself, be capable of producing a transient hysteresis that is consistent with neural inertia.

### Implications of Metastability for Brain Monitoring

The clinical utility of brain monitoring during anesthesia centers on optimizing delivery of anesthesia: giving the right amount of anesthesia to minimize the risk of awareness while minimizing side effects from excessive drug administration. Even sophisticated processing algorithms used by the BIS brain monitoring system (Medtronic Minneapolis, MN, USA) have not demonstrated superiority in preventing anesthetic awareness with recall over monitoring end-tidal anesthetic gas concentration in several large trials (Avidan et al., 2008, 2011). Why might this be?

It would be desirable for a measure that is used to titrate drug delivery to be proportional to its concentration in the brain. A reanalysis of the B-UNAWARE trial found that the BIS appeared not to vary linearly with administered gas concentration, but to have a very large region of shallow slope in the relationship between BIS and administered anesthetic concentration (Whitlock et al., 2011). One possible explanation for this is the degeneracy of the relationship between brain state and drug concentration demonstrated in rodents (Hudson et al., 2014).

For anesthetic drugs that demonstrate metastable behavior, it would be productive to understand the transition probabilities between different attractors at a given anesthetic concentration. In essence, the key problem in depth of anesthesia monitoring is the prediction of the likelihood of transitioning to a state compatible with awareness at some time in the near future. If we understand how different anesthetized states transition between each other, it should be possible to predict when interventions are needed to change the delivered dose of anesthetic. Such an approach could expand upon already existing technology to provide closed loop anesthesia for maintaining a particular burst-suppression ratio (Ching et al., 2013), which is essentially maintaining a given state transition probability.

Finally, the existence of anesthetic drugs that demonstrate metastable behavior would suggest that the simple notion of depth of anesthesia as a one-dimensional quantity, as implied by a single number from 0 to 100, should be reconsidered. The assumption of a linear scale for measuring depth of anesthesia assumes a measure of distance between the brain’s current state and wakefulness in a phase space. There is nothing wrong with attempting to derive a distance from awareness measure, but it is not clear that such a measurement is linear or even stable over time. Indeed, the idea of sufficient depth of anesthesia clearly relates to the amount of ongoing sensory stimulation, as anyone that has managed a patient in the operating room can attest. Understanding that the brain is constantly being perturbed by sensory stimulation (or “noise”), which can either be amplified by the addition of a new noxious stimulus or attenuated by, for example opiate administration, could help to integrate notions of brain state and anesthetic delivery together with intuitions from clinical care to help drive the development of more useful monitoring systems.

## Conclusions

Brain dynamics, or the transitions between different attractors in brain activity, have been understudied during anesthesia. Evidence exists for metastable attractors of brain activity during anesthesia for some model systems, and this is likely to be a ubiquitous feature of anesthetized brains, though this assumption remains to be tested. By studying the transitions between attractor structure with depths of anesthesia, it should be possible to better define a notion of distance from wakefulness in terms of brain state, which offers the opportunity to improve upon brain monitoring algorithms currently in use. Finally, identifying attractors present during unconscious states will allow further characterization of instances of prolonged or disordered recovery of consciousness.

## Author Contributions

AEH conceived the manuscript, performed a new analysis of previously published data that he had recorded with collaborators, and wrote the manuscript.

## Conflict of Interest Statement

The author declares that the research was conducted in the absence of any commercial or financial relationships that could be construed as a potential conflict of interest.
